# 3-Carb­oxy­pyridinium nitrate

**DOI:** 10.1107/S1600536812028565

**Published:** 2012-06-30

**Authors:** Khalid Al-Farhan, Miftahul Khair, Mohamed Ghazzali

**Affiliations:** a Department of Chemistry, College of Science, King Saud University, PO Box 2455, Riyadh 11451, Saudi Arabia

## Abstract

In the crystal structure of the title compound, C_6_H_6_NO_2_^+^·NO_3_^−^, the protonated cations are linked by N—H⋯O hydrogen bonds into chains along the *b* axis. The cations and anions are also linked by N—H⋯O and O—H⋯O hydrogen bonds. C—H⋯O inter­actions also occur. In the cation, the ring makes a dihedral angle of 10.1 (3)° with the carboxylate group.

## Related literature

For related structures, see: Athimoolam & Rajaram (2005 ▶); Athimoolam & Natarajan (2007 ▶); Kutoglu & Scheringer (1983 ▶); Jebas *et al.* (2006 ▶); Slouf (2001 ▶); Ye *et al.* (2010 ▶). For graph-set descriptors, see: Etter (1990 ▶); Bernstein *et al.* (1995 ▶); Motherwell *et al.* (2000 ▶).
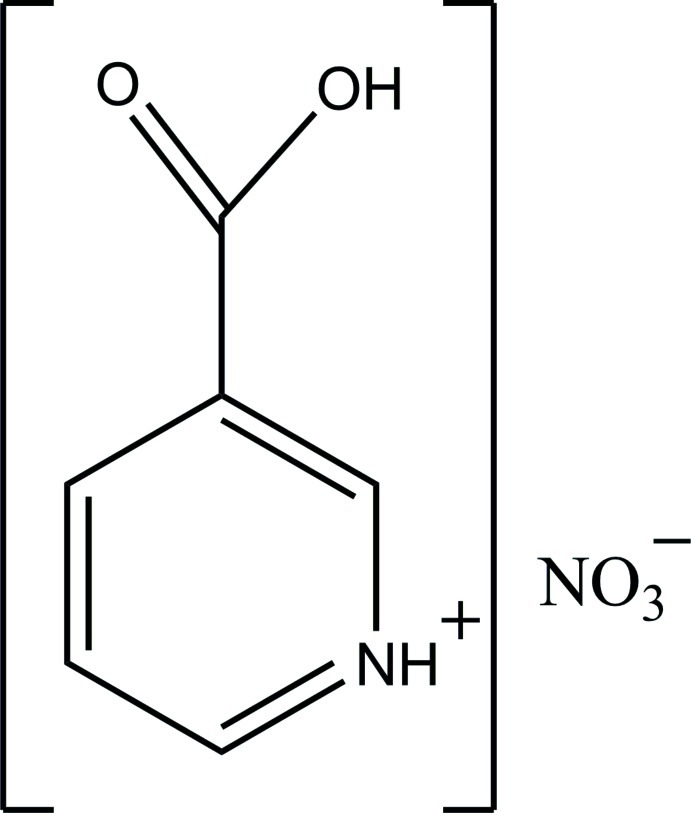


## Experimental

### 

#### Crystal data

C_6_H_6_NO_2_^+^·NO_3_^−^
*M* *_r_* = 186.13
Triclinic, 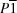

*a* = 6.7530 (4) Å
*b* = 7.5024 (4) Å
*c* = 8.4439 (5) Å
α = 81.895 (2)°
β = 82.215 (1)°
γ = 66.769 (2)°
*V* = 387.69 (4) Å^3^
*Z* = 2
Mo *K*α radiation
μ = 0.14 mm^−1^
*T* = 293 K
0.40 × 0.20 × 0.10 mm

#### Data collection

Rigaku R-AXIS RAPID diffractometer
Absorption correction: multi-scan (*CrystalClear*; Rigaku, 2007 ▶) *T*_min_ = 0.946, *T*_max_ = 0.986
15468 measured reflections
1760 independent reflections
1102 reflections with *I* > 2σ(*I*)
*R*_int_ = 0.046

#### Refinement

*R*[*F* ^2^ > 2σ(*F* ^2^)] = 0.050
*wR*(*F* ^2^) = 0.145
*S* = 1.13
1760 reflections
127 parameters
H atoms treated by a mixture of independent and constrained refinement
Δρ_max_ = 0.19 e Å^−3^
Δρ_min_ = −0.35 e Å^−3^

### 

Data collection: *CrystalClear* (Rigaku, 2007 ▶); cell refinement: *CrystalClear*; data reduction: *CrystalClear*; program(s) used to solve structure: *SHELXS97* (Sheldrick, 2008 ▶); program(s) used to refine structure: *SHELXL97* (Sheldrick, 2008 ▶); molecular graphics: *DIAMOND* (Brandenburg, 2004 ▶); software used to prepare material for publication: *publCIF* (Westrip, 2010 ▶).

## Supplementary Material

Crystal structure: contains datablock(s) I, global. DOI: 10.1107/S1600536812028565/lx2240sup1.cif

Structure factors: contains datablock(s) I. DOI: 10.1107/S1600536812028565/lx2240Isup2.hkl

Supplementary material file. DOI: 10.1107/S1600536812028565/lx2240Isup3.cml

Additional supplementary materials:  crystallographic information; 3D view; checkCIF report

## Figures and Tables

**Table 1:** Hydrogen-bond geometry (Å, °)

*D*—H⋯*A*	*D*—H	H⋯*A*	*D*⋯*A*	*D*—H⋯*A*
O1—H1*O*⋯O4^i^	0.92 (3)	1.67 (3)	2.5833 (19)	169 (2)
N1—H1*N*⋯O2^ii^	0.96 (3)	2.08 (3)	2.824 (2)	133 (2)
N1—H1*N*⋯O4	0.96 (3)	2.12 (3)	2.921 (2)	139 (2)
C3—H3⋯O3	0.93	2.45	3.330 (3)	158
C6—H6⋯O5^iii^	0.93	2.37	3.259 (2)	160
C5—H5⋯O3^iv^	0.93	2.47	3.142 (2)	129

Symmetry codes: (i) 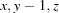; (ii) 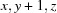; (iii) 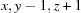; (iv) 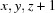.

**Fig. 1.**
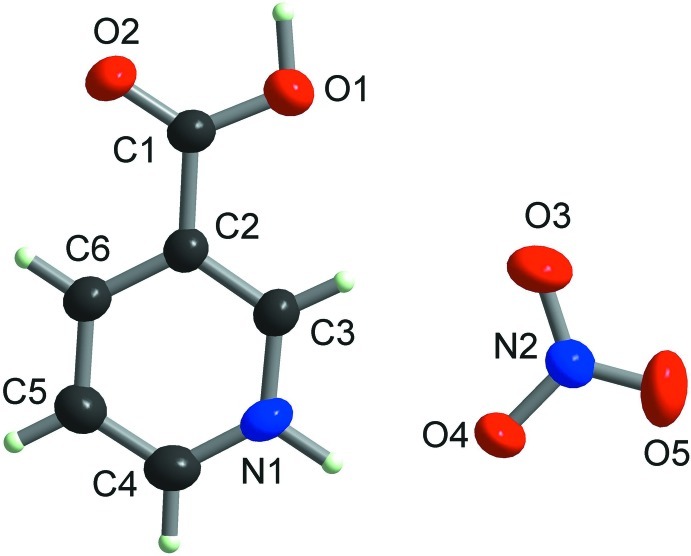
The molecular structure of the title compound with the atom numbering scheme. Displacement ellipsoids are drawn at the 50% probability level. H atoms are presented as small spheres of arbitrary radius.

**Fig. 2.**
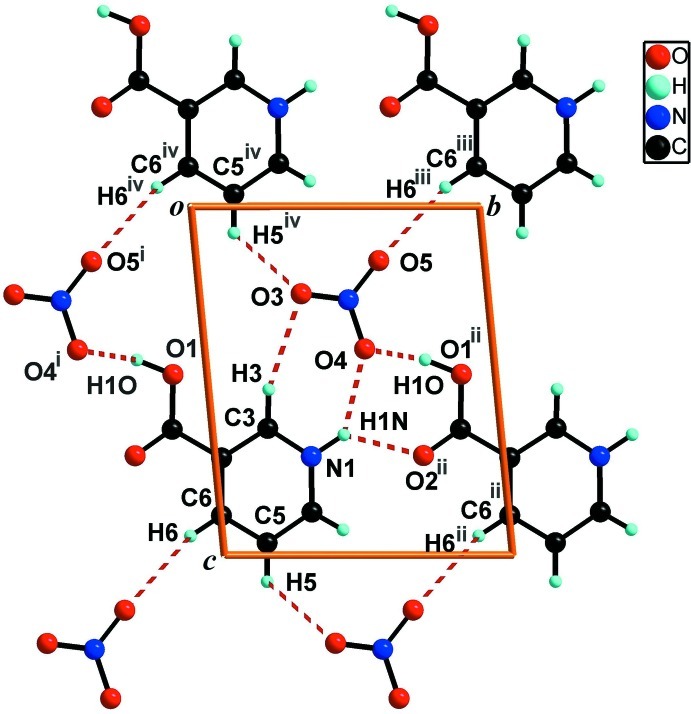
A view of the O—H···O, N—H···O and C—H···O interactions (dotted lines) in the crystal structure of the title compound. [Symmetry codes: (i) x,y - 1, z; (ii) x, y + 1, z; (iii) x,y + 1, z - 1; (iv) x, y, z - 1.]

## supplementary crystallographic information

### Comment

Previously, the crystal structures of nicotinium derivatives containing diverse anions have been reported (Athimoolam & Rajaram, 2005; Athimoolam & Natarajan, 2007; Kutoglu & Scheringer, 1983; Jebas *et al.*, 2006; Slouf, 2001; Ye *et al.*, 2010). We report herein the crystal structure of the title compound.

In the title molecule (Fig. 1), the nicotinium cation is planar with a maximum deviation for the carboxylate oxygen atom (O1) being 0.265 (2) Å. In the crystal structure (Fig. 2), the cations are linked by N—H···O hydrogen bonds (Table 1) into infinite chains along the [010] vector. Regarding the graph set descriptors (Etter, 1990; Bernstein *et al.*, 1995; Motherwell *et al.*, 2000), this N—H···O chain motif is described as C(6). The bifurcated N—H···O with O—H···O interactions (Table 1) are connecting the nicotinium with nitrates, thus defining a third-level discrete D^3^_3_(13) hydrogen bond motif in the *bc*-plane (Fig. 2).

### Experimental

The title compound was unintentionally obtained during a microwave irradiation (300 W, 150 *^o^*C, 10 min., MicroSynth, Milestone) reaction of Ce(OH)_4_ in diluted nitric acid with aqueous solution of nicotinic acid. After cooling, the solution was left undisturbed and colourless crystals were collected by filtration after one week.

### Refinement

Aromatic carbon–bound H–atoms were placed in ideal calculated positions [C—H 0.93 Å, *U*_iso_(H) = 1.2*U*_eq_(C)] and refined as riding atoms. Amine and hydroxyl hydrogen atoms were located from difference Fourier map and refined freely.

### Crystal data

**Table:**

C_6_H_6_NO_2_^+^·NO_3_^−^	*Z* = 2
*M**_r_* = 186.13	*F*(000) = 192
Triclinic, *P*1	*D*_x_ = 1.594 Mg m^−^^3^
Hall symbol: -P 1	Mo *K*α radiation, λ = 0.71075 Å
*a* = 6.7530 (4) Å	Cell parameters from 963 reflections
*b* = 7.5024 (4) Å	θ = 3.3–27.5°
*c* = 8.4439 (5) Å	µ = 0.14 mm^−^^1^
α = 81.895 (2)°	*T* = 293 K
β = 82.215 (1)°	Block, colourless
γ = 66.769 (2)°	0.40 × 0.20 × 0.10 mm
*V* = 387.69 (4) Å^3^	

### Data collection

**Table:**

Rigaku R-AXIS RAPID diffractometer	1760 independent reflections
Radiation source: fine-focus sealed tube	1102 reflections with *I* > 2σ(*I*)
Graphite monochromator	*R*_int_ = 0.046
Detector resolution: 10.0 pixels mm^-1^	θ_max_ = 27.5°, θ_min_ = 3.3°
ω scans	*h* = −8→8
Absorption correction: multi-scan (*CrystalClear*; Rigaku, 2007)	*k* = −9→9
*T*_min_ = 0.946, *T*_max_ = 0.986	*l* = −10→10
15468 measured reflections	

### Refinement

**Table:**

Refinement on *F*^2^	Primary atom site location: structure-invariant direct methods
Least-squares matrix: full	Secondary atom site location: difference Fourier map
*R*[*F*^2^ > 2σ(*F*^2^)] = 0.050	Hydrogen site location: difference Fourier map
*wR*(*F*^2^) = 0.145	H atoms treated by a mixture of independent and constrained refinement
*S* = 1.13	*w* = 1/[σ^2^(*F*_o_^2^) + (0.0739*P*)^2^ + 0.025*P*] where *P* = (*F*_o_^2^ + 2*F*_c_^2^)/3
1760 reflections	(Δ/σ)_max_ < 0.001
127 parameters	Δρ_max_ = 0.19 e Å^−^^3^
0 restraints	Δρ_min_ = −0.35 e Å^−^^3^

### Special details

**Table:**

Geometry. All esds (except the esd in the dihedral angle between two l.s. planes) are estimated using the full covariance matrix. The cell esds are taken into account individually in the estimation of esds in distances, angles and torsion angles; correlations between esds in cell parameters are only used when they are defined by crystal symmetry. An approximate (isotropic) treatment of cell esds is used for estimating esds involving l.s. planes.
Refinement. Refinement of F^2^ against ALL reflections. The weighted R-factor wR and goodness of fit S are based on F^2^, conventional R-factors R are based on F, with F set to zero for negative F^2^. The threshold expression of F^2^ > 2sigma(F^2^) is used only for calculating R-factors(gt) etc. and is not relevant to the choice of reflections for refinement. R-factors based on F^2^ are statistically about twice as large as those based on F, and R- factors based on ALL data will be even larger.

### Fractional atomic coordinates and isotropic or equivalent isotropic displacement parameters (Å^2^)

**Table:**

	*x*	*y*	*z*	*U*_iso_*/*U*_eq_	
O1	0.2671 (2)	−0.1191 (2)	0.48174 (17)	0.0528 (4)	
H1O	0.277 (4)	−0.232 (4)	0.443 (3)	0.079 (8)*	
N1	0.2399 (3)	0.3328 (2)	0.7169 (2)	0.0461 (5)	
H1N	0.245 (4)	0.449 (4)	0.654 (3)	0.086 (9)*	
C1	0.2321 (3)	−0.1404 (3)	0.6385 (2)	0.0405 (5)	
O2	0.2000 (3)	−0.2778 (2)	0.71433 (17)	0.0570 (5)	
N2	0.2859 (3)	0.5132 (2)	0.2675 (2)	0.0461 (4)	
C2	0.2350 (3)	0.0215 (2)	0.7207 (2)	0.0366 (4)	
O3	0.2861 (3)	0.3523 (2)	0.2502 (2)	0.0723 (6)	
C3	0.2385 (3)	0.1913 (3)	0.6360 (2)	0.0416 (5)	
H3	0.2399	0.2079	0.5247	0.050*	
O4	0.2781 (3)	0.5540 (2)	0.40863 (18)	0.0698 (5)	
C4	0.2393 (3)	0.3167 (3)	0.8764 (3)	0.0483 (5)	
H4	0.2410	0.4182	0.9273	0.058*	
C5	0.2361 (3)	0.1507 (3)	0.9645 (2)	0.0497 (5)	
H5	0.2348	0.1381	1.0758	0.060*	
O5	0.2917 (3)	0.6324 (3)	0.1555 (2)	0.0817 (6)	
C6	0.2350 (3)	0.0018 (3)	0.8862 (2)	0.0439 (5)	
H6	0.2341	−0.1126	0.9449	0.053*	

### Atomic displacement parameters (Å^2^)

**Table:**

	*U*^11^	*U*^22^	*U*^33^	*U*^12^	*U*^13^	*U*^23^
O1	0.0802 (11)	0.0451 (9)	0.0395 (9)	−0.0309 (8)	0.0008 (7)	−0.0094 (6)
N1	0.0598 (11)	0.0313 (9)	0.0518 (11)	−0.0232 (8)	−0.0029 (8)	−0.0032 (7)
C1	0.0501 (11)	0.0346 (10)	0.0389 (11)	−0.0185 (9)	−0.0027 (8)	−0.0052 (8)
O2	0.0937 (12)	0.0416 (8)	0.0482 (9)	−0.0412 (8)	−0.0003 (8)	−0.0045 (7)
N2	0.0578 (11)	0.0445 (10)	0.0380 (9)	−0.0205 (8)	−0.0065 (7)	−0.0053 (7)
C2	0.0447 (10)	0.0304 (9)	0.0364 (10)	−0.0171 (8)	−0.0010 (8)	−0.0033 (7)
O3	0.1120 (14)	0.0623 (11)	0.0602 (11)	−0.0464 (10)	−0.0100 (9)	−0.0208 (8)
C3	0.0533 (12)	0.0346 (10)	0.0390 (10)	−0.0202 (9)	−0.0018 (8)	−0.0025 (8)
O4	0.1353 (15)	0.0522 (10)	0.0359 (9)	−0.0481 (10)	−0.0118 (9)	−0.0077 (7)
C4	0.0580 (13)	0.0421 (11)	0.0514 (13)	−0.0237 (10)	−0.0038 (9)	−0.0126 (9)
C5	0.0660 (14)	0.0477 (12)	0.0408 (12)	−0.0264 (11)	−0.0052 (10)	−0.0077 (9)
O5	0.1135 (15)	0.0740 (12)	0.0513 (10)	−0.0380 (11)	−0.0075 (9)	0.0208 (9)
C6	0.0585 (12)	0.0352 (10)	0.0419 (11)	−0.0230 (9)	−0.0024 (9)	−0.0031 (8)

### Geometric parameters (Å, º)

**Table:**

O1—C1	1.311 (2)	N2—O4	1.261 (2)
O1—H1O	0.92 (3)	C2—C3	1.377 (2)
N1—C4	1.335 (3)	C2—C6	1.385 (3)
N1—C3	1.344 (3)	C3—H3	0.9300
N1—H1N	0.96 (3)	C4—C5	1.364 (3)
C1—O2	1.213 (2)	C4—H4	0.9300
C1—C2	1.489 (3)	C5—C6	1.379 (3)
N2—O5	1.214 (2)	C5—H5	0.9300
N2—O3	1.236 (2)	C6—H6	0.9300
			
C1—O1—H1O	107.7 (16)	N1—C3—C2	118.93 (18)
C4—N1—C3	123.13 (17)	N1—C3—H3	120.5
C4—N1—H1N	120.2 (16)	C2—C3—H3	120.5
C3—N1—H1N	116.7 (16)	N1—C4—C5	119.72 (18)
O2—C1—O1	124.77 (18)	N1—C4—H4	120.1
O2—C1—C2	121.11 (18)	C5—C4—H4	120.1
O1—C1—C2	114.11 (16)	C4—C5—C6	119.0 (2)
O5—N2—O3	123.04 (18)	C4—C5—H5	120.5
O5—N2—O4	119.22 (18)	C6—C5—H5	120.5
O3—N2—O4	117.74 (17)	C5—C6—C2	120.39 (18)
C3—C2—C6	118.80 (17)	C5—C6—H6	119.8
C3—C2—C1	121.64 (17)	C2—C6—H6	119.8
C6—C2—C1	119.56 (16)		

### Hydrogen-bond geometry (Å, º)

**Table:**

*D*—H···*A*	*D*—H	H···*A*	*D*···*A*	*D*—H···*A*
O1—H1*O*···O4^i^	0.92 (3)	1.67 (3)	2.5833 (19)	169 (2)
N1—H1*N*···O2^ii^	0.96 (3)	2.08 (3)	2.824 (2)	133 (2)
N1—H1*N*···O4	0.96 (3)	2.12 (3)	2.921 (2)	139 (2)
C3—H3···O3	0.93	2.45	3.330 (3)	158
C6—H6···O5^iii^	0.93	2.37	3.259 (2)	160
C5—H5···O3^iv^	0.93	2.47	3.142 (2)	129

Symmetry codes: (i) *x*, *y*−1, *z*; (ii) *x*, *y*+1, *z*; (iii) *x*, *y*−1, *z*+1; (iv) *x*, *y*, *z*+1.
